# Antimicrobial resistance genes and associated mobile genetic elements in Lactobacillales from various sources

**DOI:** 10.3389/fmicb.2023.1281473

**Published:** 2023-11-17

**Authors:** Eszter Kaszab, Levente Laczkó, Gábor Kardos, Krisztián Bányai

**Affiliations:** ^1^HUN-REN Veterinary Medical Research Institute, Budapest, Hungary; ^2^One Health Institute, Faculty of Health Sciences, University of Debrecen, Debrecen, Hungary; ^3^National Laboratory of Infectious Animal Diseases, Antimicrobial Resistance, Veterinary Public Health and Food Chain Safety, Veterinary Medical Research Institute, Budapest, Hungary; ^4^HUN-REN-DE Conservation Biology Research Group, Debrecen, Hungary; ^5^National Public Health Center, Budapest, Hungary; ^6^Department of Gerontology, Faculty of Health Sciences, University of Debrecen, Debrecen, Hungary; ^7^Department of Pharmacology and Toxicology, University of Veterinary Medicine, Budapest, Hungary

**Keywords:** Lactobacillales, antimicrobial resistance genes, one health, iMGE, plasmid

## Abstract

Lactobacillales are commonly used in food products and as probiotics in animal and human medicine. Despite being generally recognized as safe, lactic acid bacteria may harbor a variety of antimicrobial resistance genes (ARGs), which may be transferable to human or veterinary pathogens, thus, may pose veterinary and public health concerns. This study investigates the resistome of Lactobacillales. A total of 4,286 whole-genome sequences were retrieved from NCBI RefSeq database. We screened ARGs in whole genome sequences and assessed if they are transmissible by plasmid transfer or by linkage to integrative mobile genetic elements. In the database, 335 strains were found to carry at least one ARG, and 194 strains carried at least one potentially transferable ARG. The most prevalent transferable ARG were *tet*M and *tet*W conferring antibiotic resistance to tetracycline. This study highlights the importance of the One Health concept by demonstrating the potential for Lactobacillales, commonly used in food products, to serve as reservoirs and vectors for ARGs.

## 1 Introduction

Lactic acid bacteria (LAB) are frequent and abundant members of the human and animal microbiota and at the same time have a long history of utilization by humans for various purposes. Fermented food and beverages are ancient, and their versatility is considerable. Furthermore, many of these are considered to carry health benefits due to favorable nutritional properties and pathogen exclusion. Utilization of fermented or putrified food has been documented as early as more than 8,000 years ago (Boethius, 2016), and it is widely hypothesized based on archeological and evolutionary evidence that their preference predated the appearance of modern humans (Amato et al., 2021).

In addition, the traditional wisdom that such food fermented by lactic acid bacteria are beneficial for health incited their usage as probiotics, rapidly gaining popularity in nutrition as well as in medicine. Studies have shown that LAB supplementation can have a positive impact on various health outcomes, including gastrointestinal health, immune function, and even mental health (Marco et al., 2017). Therefore Hill et al. (2014) reworked the definition of probiotics to the following: “live microorganisms which when administered in adequate amounts confer a health benefit on the host.” The International Scientific Association for Probiotics and Prebiotics (ISAPP) recommends that the term probiotic be used only on products that deliver live microorganisms with a suitable viable count of well-defined strains with a reasonable expectation of delivering benefits for the wellbeing of the host (Hill et al., 2014). However, it is important to note that the effects of LAB can vary depending on the specific strain and the individual’s health status. Probiotics are recommended widely by doctors and pharmacists and have gained fame and popularity in the general public, which brought about their increasing consumption in human as well as in veterinary medicine (Sharma et al., 2014). Overall, LAB play a crucial role in the development of food products with living flora, and are thought to be a component of a healthy diet due to their potential health benefits. The size of the probiotic market is estimated at $65 billion and is projected to increase (Abid and Koh, 2019), despite that measurable health benefit has been proven only in case of few of the many indications in which they are widely used (Abid and Koh, 2019). This increase anticipates the addition of novel strains to the probiotic arsenal, most of which is expected to come from the group of LAB.

Lactic acid bacteria have been shown to harbor acquired antibiotic resistance genes (Sharma et al., 2014). Considering their frequent association with the microbiota of humans and animals, playing a role in the food chain as live components of fermented food together with the frequency of probiotic administration both to humans and to animals, these bacteria have ample opportunity to serve as important sources, vehicles, and targets for exchanging mobile genetic elements, including those harboring antibiotic resistance genes. Thus, they may infest the microbiome we intend to improve using them with antibiotic resistance genes (Montassier et al., 2021). Ironically, probiotics are also frequently recommended to diminish side effects of taking antibiotics (Ouwehand et al., 2016), and frequently prescribed together with them (Rodgers et al., 2013) or to spare them.

Though antibiotic resistance among commercial probiotic products is started to be surveyed (Wong et al., 2015; Cui et al., 2020; Rozman et al., 2020; Toth et al., 2021), approaches for comprehensive understanding of the resistance gene array occurring in LAB, thus an overview of the potential threats they pose, are scarce. This study aims at reviewing the genomes of LAB deposited in the NCBI reference sequence database in order to determine the abundance and distribution of resistance genes in the available lactic acid bacterium genomes.

## 2 Materials and methods

### 2.1 Data

We screened publicly available reference genomes of Lactobacillales for the presence and identity of antimicrobial resistance genes (ARG). Genome sequences were obtained from the NCBI RefSeq, a well-curated database of high-quality reference genome sequences. All accessions of Lactobacillales (*n* = 4,286) were downloaded on 04/10/2021, including the genome sequences in fasta format and the corresponding metadata (Supplementary Table S1). Accessions were divided into nine categories by their isolation source to make the interpretation of results easier (animals and humans, animal source food, dairy products, foods and beverages of plant origin, plant and environment, other).

### 2.2 Bioinformatic analysis

Mass screening of individual genomes was achieved using ABRicate (Seemann, 2020; https://github.com/tseemann/abricate) which software relies on BLAST (Altschul et al., 1990) to match the nucleotide sequences of ARGs against genome sequences. ABRicate used the CARD database (database version 3.1.4, 2021-10-05; McArthur et al., 2013) to detect acquired ARGs in the genome sequences. Contigs bearing at least one ARG were screened for integrative mobile genetic elements (iMGE) using MobileElementFinder 1.0.5 (Johansson et al., 2021). Then, we checked the distance between the coordinates of ARGs and iMGEs located on the same contig and defined them as linked if their distance was less than 10,000 base pairs (bps). The average gene length of prokaryotes is approximately 1,000 bp. We hypothesized that there is a high probability of linkage if a ARG can be found within a maximum distance of about 10 ORF distance (*ca.* 10,000 bp). We modified the concept of Johansson et al. (2021), according to which ARGs are considered linked to AMRs if they can be found within a distance defined by the length of the longest iMGE. Our threshold is more conservative to avoid identifying false positives. Toth et al. (2021), also following the concept of Johansson et al. (2021) found this threshold for *Lactococcus* to be 11,256 bps, which is close to our universal threshold defined for the sake of simplicity. We acknowledge that a threshold-based linkage assessment may not accurately describe the actual molecular events, but argue that in this case the majority of ARGs linked to iMGEs would be identified. The threshold of 10,000 bps is about twice longer the average length of all identified iMGEs (4260.4 bps) and is 25% of the length of the longest iMGE (52,209 bps). Accessions were subject to phylogenetic reconstruction to evaluate if the presence/absence of ARGs is condensed in the phylogeny of Lactobacillales. First, we created a core alignment of samples. We obtained the amino acid sequences of 402 genes characteristic of Lactobacillales from the BUSCO (version 5.2.2; Simao et al., 2015) single-copy orthologous gene database (odb10). Then, we used tblastn 2.10+ (Altschul et al., 1990) to match each core gene amino acid sequence against the database of available Lactobacillales genomes created with makeblastdb 2.10+ (Altschul et al., 1990). The best hits with a query coverage larger than 90% and an identity higher than 85% were retained and aligned using MAFFT 7.490 (Katoh et al., 2002) with the --auto option turned on. We used AMAS.py 1.0 (Borowiec, 2016) to concatenate the gene alignments and to retrieve alignment statistics. Then, the accession’s phylogenetic relationships were reconstructed with FastTree 2.1.11 (Price et al., 2010), explicitly designed for large alignments, using default values. Phylogenetic trees were rooted using the minimal ancestor deviation method as implemented in MAD 2.2 (Wade et al., 2020). In the next step, we reconstructed the phylogeny using the abovementioned methods of genome accessions harboring at least on ARG. The phylogenetic tree and the distribution of ARGs with linked iMGEs were visualized using the ggplot2 3.3.5 (Wickham, 2016) and ggtreeExtra 1.0.4 (Xu et al., 2021) R 4.0.4 packages (R Core Team, 2022). The classification of genera followed the guidelines of Zheng et al. (2020). To evaluate the chance of spreading ARGs, the plasmid origin of contigs with ARGs linked to iMGEs was predicted by using PlasFlow 1.1 (Krawczyk et al., 2018).

## 3 Results

### 3.1 ARG diversity in Lactobacillales

**Figure 1:**
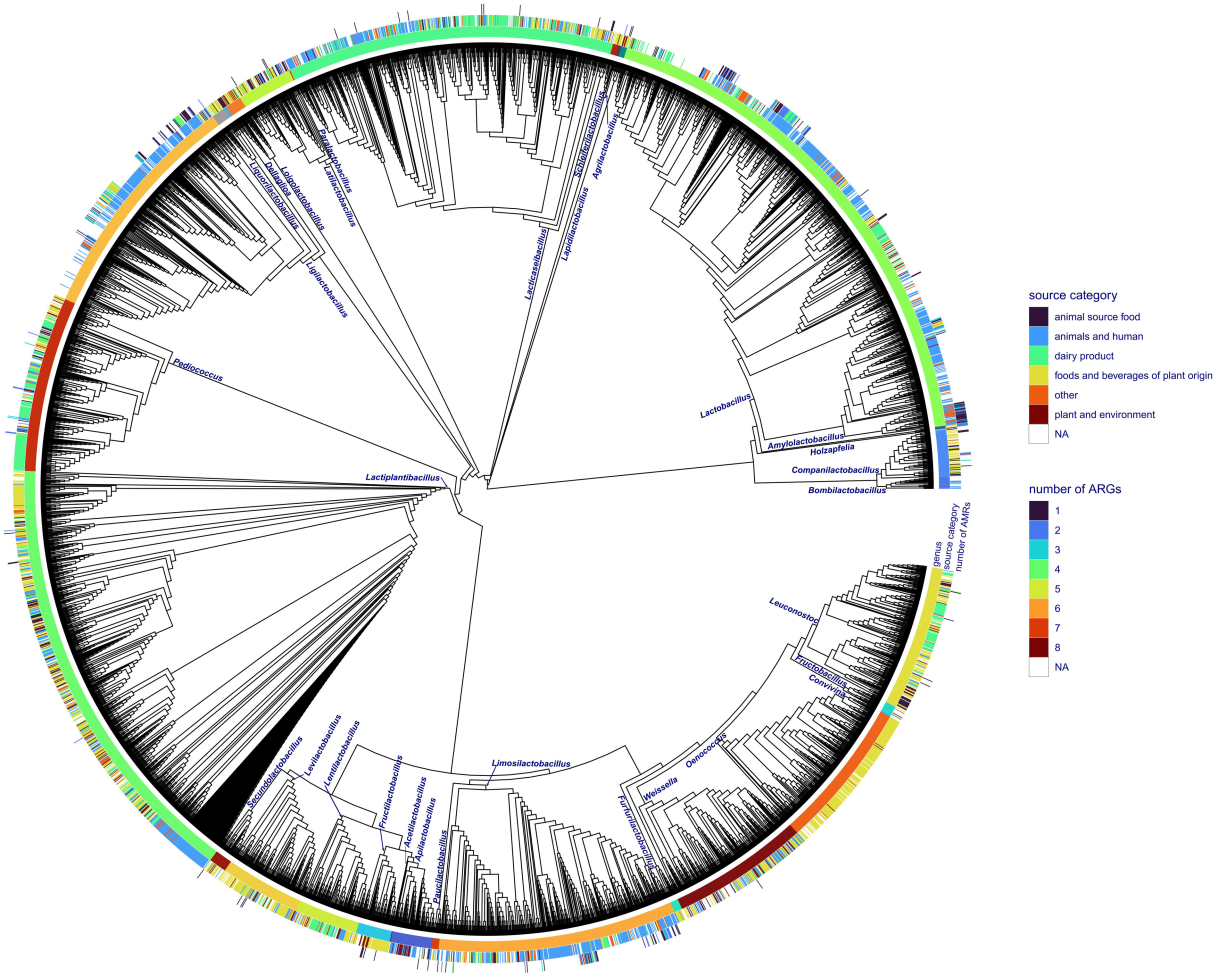
Phylogenetic reconstruction of all the Lactobacillales genome accessions obtained from NCBI RefSeq with the corresponding isolation source category and number of ARGs discovered in particular genomes. The phylogenetic tree is shown as a cladogram.

**Figure 2:**
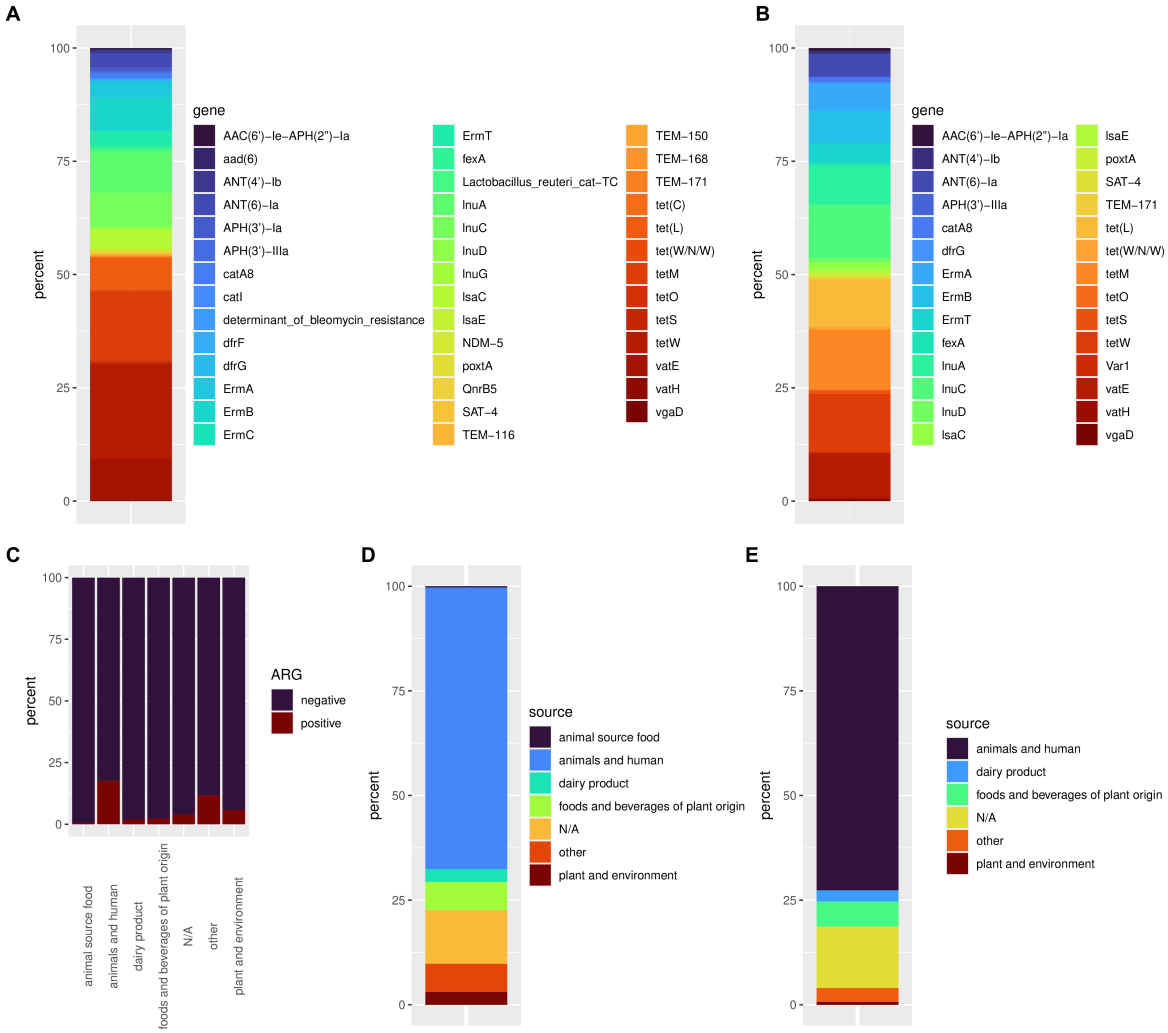
The relative frequency of all discovered ARGs **(A)** and the relative frequency of ARGs associated with iMGEs **(B)**. Ratio of ARG positive samples by isolations sources **(C)**, isolation source frequency of accessions showing at least one ARG **(D)**, and accessions showing ARGs linked to iMGEs **(E)**.

Out of 4,286 available genomes, 334 (7.8%) harbored at least one ARG. In total, we discovered 42 different ARGs in accessions of Lactobacillales. Accessed Lactobacillales genomes contained up to eight ARGs (Figure 1). The *tet*W gene conferring antibiotic resistance to tetracycline showed the highest frequency with 151 occurrences, followed by *tet*M harbored by 109 accessions (Supplementary Tables S1, S2; Figure 2A). The genes *lnu*A, *lnu*C, and *lsa*C conferring resistance to lincosamides; *Erm*A, *Erm*B, and *Erm*T conferring antibiotic resistance to lincosamides and macrolides; *ANT*(6)-Ia conferring resistance to streptomycin; *vat*E conferring resistance to dalfopristin and *tet*(L) conferring resistance to tetracyline, could be found in a moderate number of genomes (*n* = 22–65). The remaining ARGs were found in less than 10 accessions (Supplementary Table S2). We discovered 193 accessions harboring ARGs linked to iMGEs. The *tet*M and *tet*W gene were associated with an iMGE in 40 and 39 accessions, respectively. The genes *Inu*C, *tet*(L), and *vat*E were associated with iMGEs in more than 30 Lactobacillales genomes. The resistance genes *Inu*A, *Erm*B, *Erm*A, *ANT*(6)-Ia, and *Erm*T were found on iMGE-bearing contigs in a moderate number of accessions (*n* = 13–26). The rest of the ARGs (Figure 1; Supplementary Figure S1B) were linked to iMGEs in up to five genomes (Supplementary Table S3; Figure 2B). The most common ARG associations were *tet*W, *tet*M, *Erm*B, and ANT(6)-Ia. Some genomes contained multiple iMGE associated ARGs, for instance *Latilactobacillus sakei*, *Ligilactobacillus salivarius*, and *Lactobacillus amylovorus* genomes among others.

### 3.2 ARG abundance by source of isolation

Antimicrobial resistance genes could be discovered in all isolation sources (Table 1). The highest number and ratio of ARG positive genomes (17.6%) could be linked to isolation source categories animals and humans (Table 1; Figures 2C,D). Samples isolated from dairy products, food, and beverages of plant origin, plants and the environment showed a lower ratio of ARG positive accessions ranging from 0.6 to 5.8%. We discovered only one genome (*Ligilactobacillus sali*var*ius*, isolation source: ground beef) harboring an ARG isolated from food of animal origin. Almost 4% of ARG positive genome sequences had an unknown isolation source, and 11.8% of such accessions could not be associated with the isolation source categories defined in this study. Similarly, bacterial genomes from animals and humans showed the highest frequency of iMGE-associated ARGs (Table 1; Figure 2E), followed by foods and beverages of plant origin and dairy products. The only ARG identified from food of animal origin (0.6% of accessions in the category) was not linked to iMGEs. Over 14% of iMGE positive accessions had an unknown origin, and 3.3% of such genomes were associated with the “other” isolation source.

**Table 1:** Frequency of ARG positive samples by different isolation sources.

Isolation source	Total accessions	ARG positive accessions (%)	Accessions with ARGs linked to iMGEs (%)
N/A	1,069	49 (4.5)	22 (2.1)
Animals and human	1,247	220 (17.6)	109 (8.7)
Animal source food	167	1 (0.6)	0 (0)
Dairy product	515	10 (1.9)	4 (0.8)
Foods and beverages of plant origin	929	22 (2.4)	9 (0.9)
Other	186	22 (11.8)	5 (2.7)
Plant and environment	173	10 (5.8)	1 (0.6)

### 3.3 ARGs in different taxa of Lactobacillales

The core gene amino acid alignment of all genomes consisted of 207,592 positions (Supplementary Table S4) with 18.6% of missing characters. The alignment contained 166,530 variable sites, of which 156,870 appeared to be informative. The phylogenetic reconstruction of the whole dataset (Figure 2) resulted in a well-resolved phylogenetic tree. All internal branches leading to distinct genera received statistical support higher than 95%, except the branch separating *Lactiplantibacillus* from *Pediococcus* (SH-like support value, 78%).

The core gene alignment of ARG harboring accessions showed 139,132 variable and 132,339 informative sites out of 197,279 positions. This alignment lacked 13.889% of positions (Supplementary Table S4). The phylogeny of the ARG-harboring genome accessions reconstructed using core gene alignments showed a support value higher than 99% for all the internal branches. Lower support values could only be observed for short branches separating strains within the same genus.

**Figure 3:**
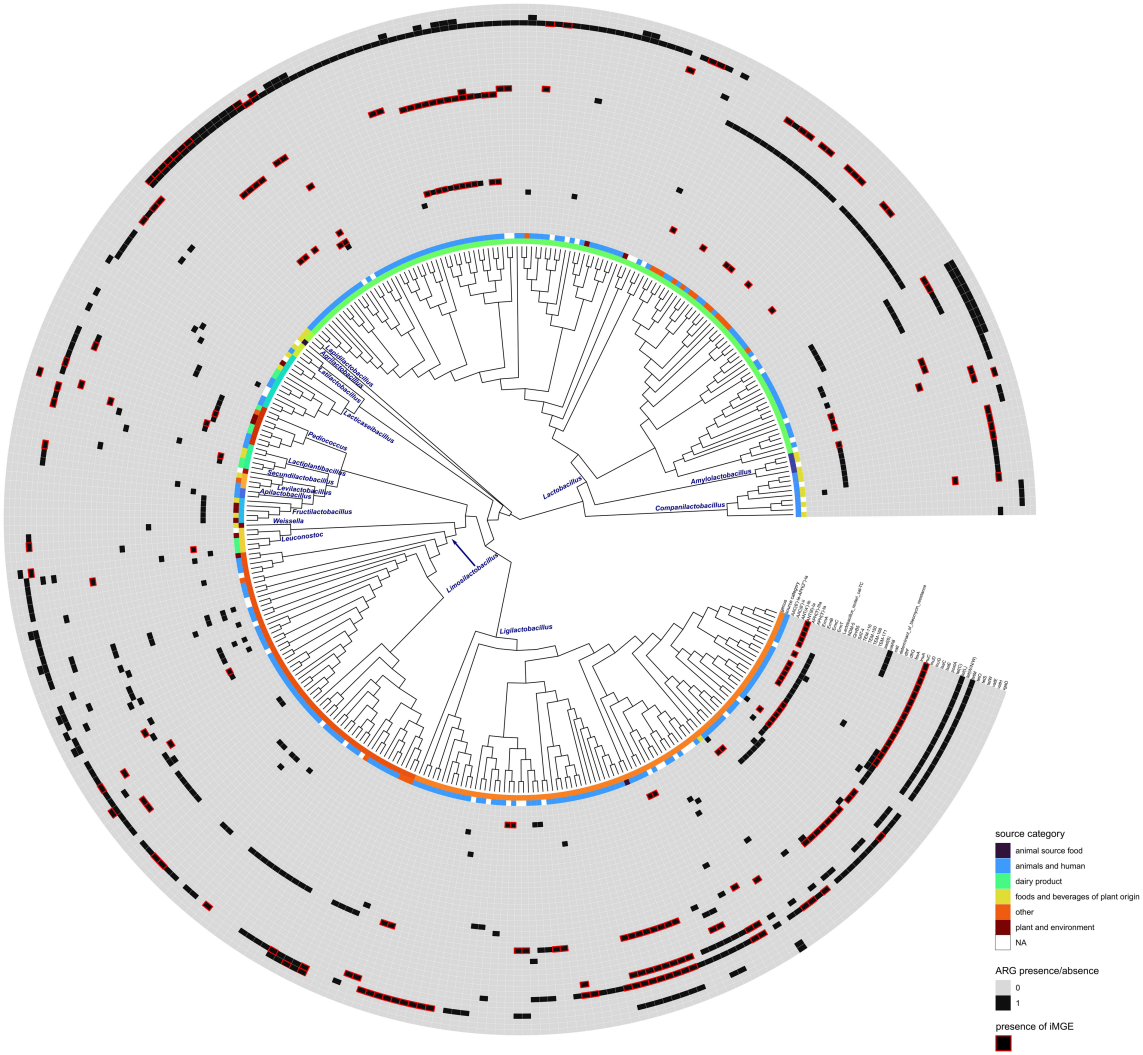
Phylogenetic reconstruction of ARG harboring genome accessions. Isolation source category and the presence of each ARG are displayed as different tracks. Red rectangles show ARGs that were found within 10,000 bps of iMGEs. The phylogenetic tree is shown as a cladogram.

Seventeen out of 31 genera showed at least one accession harboring ARGs (Figure 1). The *tet*M gene was present in the highest number of genera with the highest frequency in *Ligilactobacillus* (Supplementary Figure S1A). *Erm*B could be identified in a lower number of genera and appeared to be the most characteristic of *Lactobacillus* and *Pediococcus*. The ARGs *vat*E and *tet*W were found in six and three genera, respectively, and their frequency appeared to be relatively high in *Lactobacillus*. The rest of the ARGs could be identified in up to five genera of Lactobacillales, and some of them appeared to be rare and characteristic of certain genera (Supplementary Figure S1A; Figure 3). The *tet*M and *Erm*B genes found in several genera were frequently associated with iMGEs (Supplementary Figure S1B). Almost all *Lactobacillus* accessions harboring *Erm*T were associated with the presence of iMGEs. More than 40% of the *tet*W observations in *Lactobacillus* were linked to iMGEs and this ARG was also relatively frequent in *Ligilactobacillus*. The *Inu*C, *tet*(L), and *vat*E genes were linked to iMGEs in *Lactobacillus* and *Ligilactobacillus* with *vat*E occasionally occurring in *Leuconostoc*, *Ligilactobacillus*, *Limosilactobacillus*, and *Pediococcus*. *Ligilactobacillus* had a relatively high number of accessions with the ARGs *Erm*A and *ANT*(6)-Ia linked to iMGEs, of which *ANT*(6)-Ia were also observed in *Lactobacillus* and *Lactiplantibacillus* and *Companilactobacillus*. The rest of the ARGs were much more rarely associated with iMGEs (Supplementary Figure S1B; Figure 3).

PlasFlow identified 24 contigs of plasmid origin that harbored ARGs linked to iMGEs. The majority of the remaining contigs (*n* = 142) were identifiable as bacterial chromosomes alongside 27 unidentified contigs (i.e., neither identifiable as plasmids nor as chromosomes). For the sake of simplicity, below, we refer to this category as bacterial chromosomes. When located on a plasmid, ARGs were associated with up to four iMGEs and up to 10 iMGEs when located on a bacterial chromosome. One frequently discovered ARG, *tet*W was associated with iMGEs on bacterial chromosomes. Some genes were exclusive to plasmids or to chromosomes (Supplementary Figure S2). The *tet*M gene was linked to iMGEs when located on plasmids and in approximately half of the observations located on chromosomes, similarly to *tet*(L). The latter had less observations on chromosomes (Supplementary Figure S2). When located on a chromosome, *Inu*C and *Inu*A could be characterized with a high frequency of iMGEs, whereas mobile elements linked to these ARGs appeared less frequently on plasmids. The gene *Erm*T was linked to iMGEs only on bacterial chromosomes, and *ANT*(6)-Ia was associated with iMGEs with a high frequency both on chromosomes and plasmids.

## 4 Discussion

Historically, LAB are thought to be beneficial to health and have a positive effect on human and animal diet. Their fermentative metabolism has been exploited since modern humans arose and started processing animal and plant-based foods. Based on this experience many years old, fermented food is promoted and advertised with the claimed health-preserving properties of such food and the “living flora” therein (Leeuwendaal et al., 2022; Shah et al., 2023). Such claims are increasingly used to foster the multibillion dollar food and beverage industry (Abid and Koh, 2019). In addition, recommending probiotics based frequently on LAB is a common practice in humans to restore integrity of the microbiota of the gastrointestinal tract as well as in veterinary applications to achieve production advantage, sometimes without firm evidence on their utility. Members of Lactobacillales are used extensively for purposes of human and veterinary medicine and nutrition (Hill et al., 2014; Garcia-Hernandez et al., 2016; Dowarah et al., 2017; Al-Yami et al., 2022).

Besides the benefits, these bacteria may harbor ARGs and have the capacity to transfer these ARGs to other bacteria colonizing the individual or the animal receiving probiotic formulas or consuming fermented food through iMGEs. When pathogenic bacteria acquire such iMGE-linked ARGs, the risk that resistant pathogenic bacterial strains evolve significantly increases. This is especially important because these bacteria are generally recognized as safe, therefore, used excessively in human nutrition, feeds for companion animals and in animal husbandry (Montassier et al., 2021; Radovanovic et al., 2023).

Plasmid transmissibility has been studied extensively in LAB, particularly in species commonly used in food fermentation or as probiotics, such as *Lactobacillus, Lactococcus, Enterococcus*, and *Streptococcus* (Schleifer et al., 1985; Igimi et al., 1996; Ammann et al., 2008). These bacteria are known to exchange plasmids both within and between species (Bolotin et al., 2004), leading to the spread of beneficial as well as potentially harmful traits. The mechanisms of plasmid transfer can also vary depending on the specific LAB species and the type of plasmid involved (Igimi et al., 1996; Ammann et al., 2008; Ortiz Charneco et al., 2021). Such plasmid transfer events may trigger acquisition of resistance genes from the host microbiota from pathogens or commensals, which may then be transmitted, for example, in an animal flock to microbiota of other individuals, or these LAB may be the source for resistance genes.

As outlined by the results above, understanding plasmid transmissibility in LAB is important for the development of safe and effective probiotic products, as well as for the control of antibiotic resistance in foodborne pathogens (Ammor et al., 2007; van Reenen and Dicks, 2011). Such a transfer may occur from the LAB to members of the human or animal microbiota, then eventually to potential pathogens. LABs in probiotics or in food may acquire the genes from pathogens or commensals and then serve as a vector to environmental bacteria when passed to the environment. This risk is highlighted by the higher occurrence of resistance genes in LABs from human and animal sources. For the safe development of probiotics the mechanisms of plasmid transfer in LAB and the factors that influence the spread of plasmids between LAB and other bacteria in complex microbial environments and community (such as the gut microbiota) should be extensively studied (Mayo et al., 2014).

In this study, we evaluated the ARG repertoire of lactobacilli using the RefSeq database as source of annotated high-quality genomes. Although this approach may have prevented the identification of a more complex landscape of ARG repertoire by omitting a fairly large amount of data (e.g., fragmented genomes, data from metagenomic surveys, etc.), we felt that the ARG diversity in LAB of the order Lactobacillales is more than satisfactorily illustrated with the analysis of over 4,000 bacterial reference genomes, and sacrificed exhaustive analysis to avoiding use of unconfirmed and potentially misleading data in the study. In similar studies, metadata (such as host and isolation source) are of paramount importance, since the prevalence and spreading potential of ARGs can be only assessed in their presence. The accessions downloaded from RefSeq had a high proportion of missing metadata (4% for ARG-positive samples and 14% for iMGE-positive samples). This observation aligns with the findings of Yarmosh et al. (2022), who also reported a lack of metadata in RefSeq and called for the deep curation of metadata to aid the reusability of the data and increase the reproducibility of research. The imbalanced number of samples classified by their isolation source (Table 1) highlights the importance of data generation in microbial ecology to study microbial diversity (Mony et al., 2020) and the need for high-quality data sharing (see Wilkinson et al., 2016).

In addition to plasmid transmissibility, the linkage of ARGs to iMGEs can be another potential threat. Not surprisingly, Lactobacillales harboring ARG and even ARG linked to iMGEs, were 10–100 times more commonly found in isolates originating from animal and human sources than those isolated from other sources. As production animals, pets and humans are frequently treated with antibiotics, their bacteria are markedly more exposed, in general, than strains from other sources. This higher selective pressure may result in higher risk of horizontal transfer of resistance genes from human or veterinary pathogens. Probiotics are consumed frequently by animals and humans, the increased opportunity for such transfers may also be due to consumption of large amounts of these bacteria as probiotics (Imperial and Ibana, 2016; Liu et al., 2020).

These results draw attention yet again to the One Health aspects of ARGs. LAB present in fermented plant-derived food or in dairy products consumed may exchange ARGs with LAB as well as other bacteria associated with the human or animal microbiomes, possibly acting as their sources or vectors along the food chain, e.g., probiotic strains used in animal feed or their genes in other host bacteria may persist in the animal product and reach consumers. ARGs harbored in Lactobacillales warn for caution when creating and using probiotics, especially in veterinary practice where use of probiotics is expected to rise.

## Data availability statement

The original contributions presented in the study are included in the article/Supplementary material, further inquiries can be directed to the corresponding author.

## Author contributions

EK: Formal Analysis, Methodology, Writing – original draft. LL: Formal Analysis, Methodology, Visualization, Writing – original draft. GK: Conceptualization, Funding acquisition, Supervision, Writing – original draft. KB: Conceptualization, Funding acquisition, Writing – original draft.
