# Supplementary figures and images for: Antimicrobial resistance genes and associated mobile genetic elements in Lactobacillales from various sources
Source: Front Microbiol. 2023 Nov 17;14:1281473. doi: 10.3389/fmicb.2023.1281473 (PMC10690630; doi:10.3389/fmicb.2023.1281473)

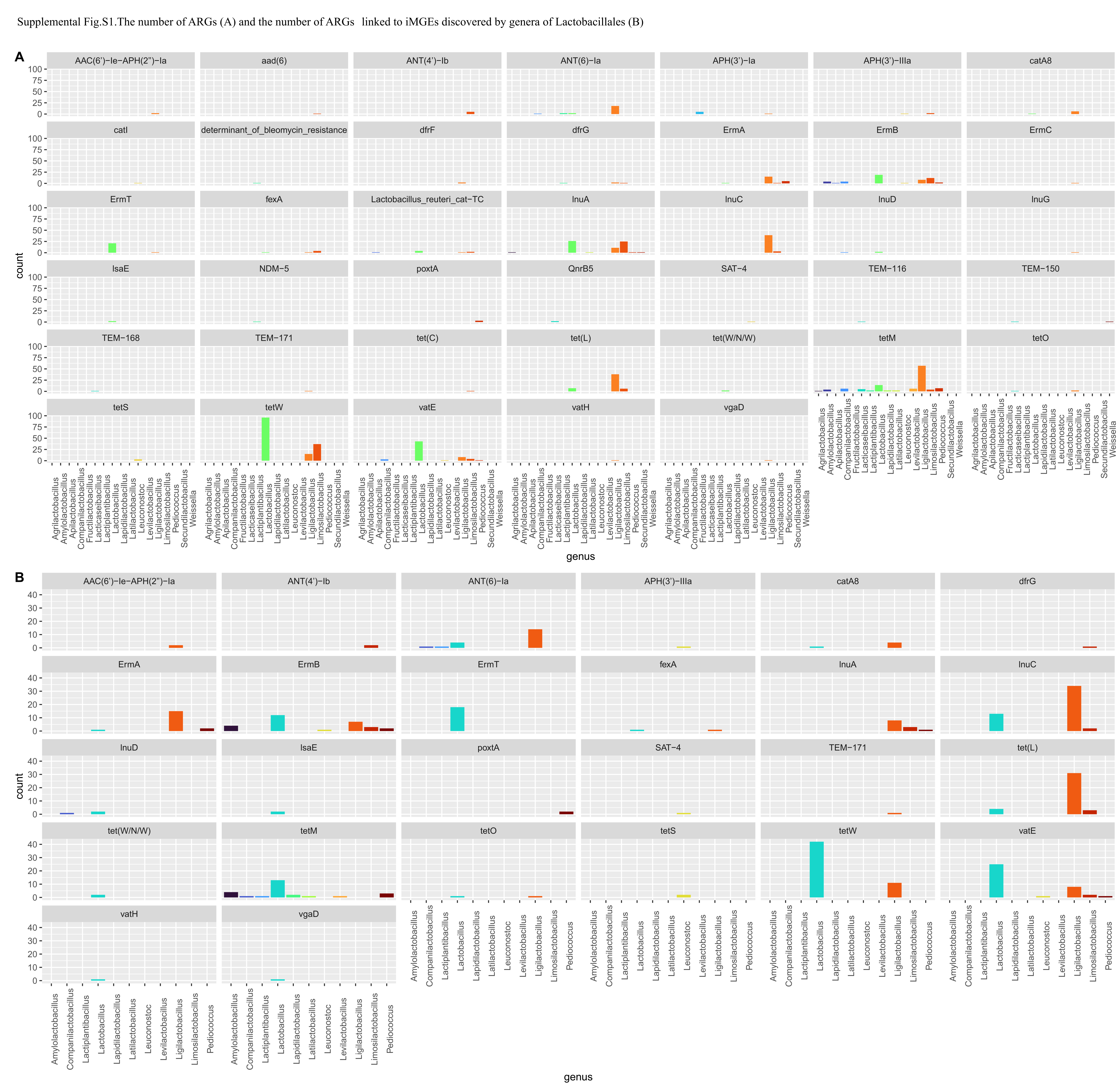

Supplement: Supplementary file 1 [file Image_1.PNG]

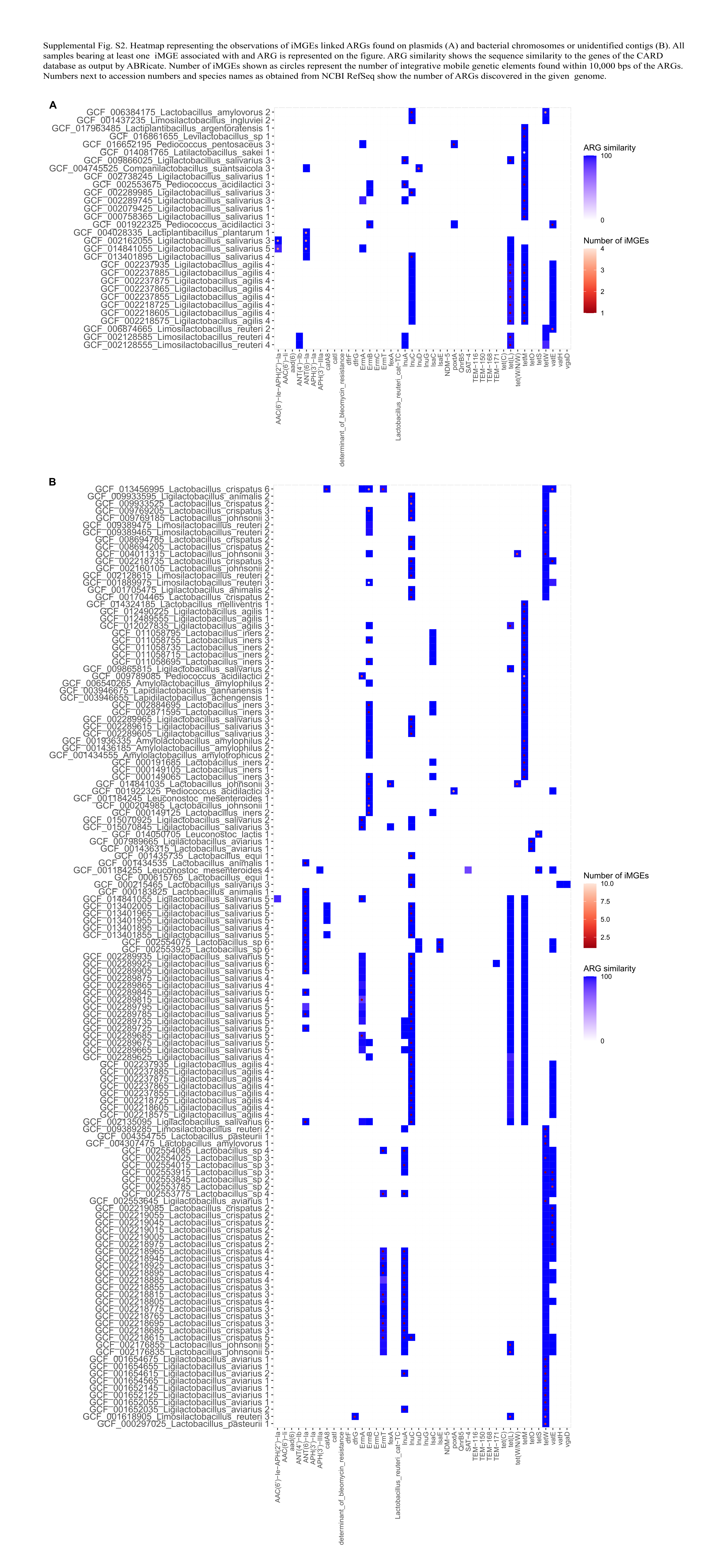

Supplement: Supplementary file 2 [file Image_2.PNG]
