# Supplementary material for: Antimicrobial resistance genes and associated mobile genetic elements in Lactobacillales from various sources
Source: Front Microbiol. 2023 Nov 17;14:1281473. doi: 10.3389/fmicb.2023.1281473 (PMC10690630; doi:10.3389/fmicb.2023.1281473)
Supplement: Supplementary file 4 [file Table_2.DOCX]

Supplementary material

Table S2. Number of accessions harboring the given ARG associated and the relative frequency of resistance genes.

| **ARG** | **Number of accessions harboring the ARG** | **Relative frequency of the given ARG (%)** |
| --- | --- | --- |
| aad(6) | 1 | 0.1394700 |
| catI | 1 | 0.1394700 |
| determinant_of_bleomycin_resistance | 1 | 0.1394700 |
| ErmC | 1 | 0.1394700 |
| lnuG | 1 | 0.1394700 |
| NDM-5 | 1 | 0.1394700 |
| SAT-4 | 1 | 0.1394700 |
| TEM-116 | 1 | 0.1394700 |
| TEM-168 | 1 | 0.1394700 |
| TEM-171 | 1 | 0.1394700 |
| tet(C) | 1 | 0.1394700 |
| vatH | 1 | 0.1394700 |
| vgaD | 1 | 0.1394700 |
| AAC(6')-Ie-APH(2'')-Ia | 2 | 0.2789400 |
| dfrF | 2 | 0.2789400 |
| lsaE | 2 | 0.2789400 |
| QnrB5 | 2 | 0.2789400 |
| TEM-150 | 2 | 0.2789400 |
| tet(W/N/W) | 2 | 0.2789400 |
| APH(3')-IIIa | 3 | 0.4184100 |
| lnuD | 3 | 0.4184100 |
| poxtA | 3 | 0.4184100 |
| tetO | 3 | 0.4184100 |
| tetS | 3 | 0.4184100 |
| dfrG | 4 | 0.5578801 |
| Lactobacillus_reuteri_cat-TC | 4 | 0.5578801 |
| ANT(4')-Ib | 5 | 0.6973501 |
| APH(3')-Ia | 6 | 0.8368201 |
| fexA | 6 | 0.8368201 |
| catA8 | 7 | 0.9762901 |
| ErmT | 22 | 3.0683403 |
| ANT(6)-Ia | 23 | 3.2078103 |
| ErmA | 23 | 3.2078103 |
| lsaC | 30 | 4.1841004 |
| tet(L) | 51 | 7.1129707 |
| ErmB | 53 | 7.3919107 |
| lnuC | 54 | 7.5313808 |
| vatE | 64 | 8.9260809 |
| lnuA | 65 | 9.0655509 |
| tetM | 109 | 15.2022315 |
| tetW | 151 | 21.0599721 |

Table S3. Number of accessions harboring the given ARG associated with at least one iMGE and the relative frequency of resistance genes.

| **ARG** | **Number of accessions harbouring the ARG** | **Relative frequency of the given ARG (%)** |
| --- | --- | --- |
| APH(3')-IIIa | 1 | 0.3300330 |
| dfrG | 1 | 0.3300330 |
| SAT-4 | 1 | 0.3300330 |
| TEM-171 | 1 | 0.3300330 |
| tetO | 1 | 0.3300330 |
| vatH | 1 | 0.3300330 |
| vgaD | 1 | 0.3300330 |
| AAC(6')-Ie-APH(2'')-Ia | 2 | 0.6600660 |
| ANT(4')-Ib | 2 | 0.6600660 |
| fexA | 2 | 0.6600660 |
| lsaE | 2 | 0.6600660 |
| poxtA | 2 | 0.6600660 |
| tetS | 2 | 0.6600660 |
| tet(W/N/W) | 2 | 0.6600660 |
| catA8 | 3 | 0.9900990 |
| lnuD | 3 | 0.9900990 |
| lsaC | 5 | 1.6501650 |
| ErmT | 13 | 4.2904290 |
| ANT(6)-Ia | 15 | 4.9504950 |
| ErmA | 18 | 5.9405940 |
| ErmB | 22 | 7.2607260 |
| lnuA | 26 | 8.5808580 |
| vatE | 30 | 9.9009900 |
| tet(L) | 32 | 10.5610561 |
| lnuC | 36 | 11.8811881 |
| tetW | 39 | 12.8712871 |
| tetM | 40 | 13.2013201 |

Table S4. Statistics of the amino acid alignment used for the phylogenetic reconstructions.

| **Data set** | **No of taxa** | **Alignment length** | **Total matrix cells** | **Undetermined characters** | **Missing percent** | **No variable sites** | **Proportion variable sites** | **Parsimony informative sites** | **Proportion parsimony informative** |
| --- | --- | --- | --- | --- | --- | --- | --- | --- | --- |
| All accessions | 4,286 | 207,592 | 8,897,393,12 | 1,656,156,32 | 18.614 | 166,530 | 0.802 | 156,870 | 0.756 |
| ARG harboring accessions | 334 | 197,279 | 6,589,118,6 | 9,151,670 | 13.889 | 139,132 | 0.705 | 132,339 | 0.671 |
